# Diagnostic Algorithm Based on Machine Learning to Predict Complicated Appendicitis in Children Using CT, Laboratory, and Clinical Features

**DOI:** 10.3390/diagnostics13050923

**Published:** 2023-03-01

**Authors:** Jieun Byun, Seongkeun Park, Sook Min Hwang

**Affiliations:** 1 Department of Radiology, College of Medicine, Ewha Womans University, Seoul 07804, Republic of Korea; 2 Machine Intelligence Laboratory, Department of Smart Automobile, Soonchunhyang University, Asan 31538, Republic of Korea; 3 Department of Radiology, Kangnam Sacred Heart Hospital, Hallym University College of Medicine, Seoul 07441, Republic of Korea

**Keywords:** computed tomography, algorithms, children, appendicitis, perforated appendicitis

## Abstract

To establish a diagnostic algorithm for predicting complicated appendicitis in children based on CT and clinical features. Methods: This retrospective study included 315 children (<18 years old) who were diagnosed with acute appendicitis and underwent appendectomy between January 2014 and December 2018. A decision tree algorithm was used to identify important features associated with the condition and to develop a diagnostic algorithm for predicting complicated appendicitis, including CT and clinical findings in the development cohort (*n* = 198). Complicated appendicitis was defined as gangrenous or perforated appendicitis. The diagnostic algorithm was validated using a temporal cohort (*n* = 117). The sensitivity, specificity, accuracy, and area under the receiver operating characteristic curve (AUC) from the receiver operating characteristic curve analysis were calculated to evaluate the diagnostic performance of the algorithm. Results: All patients with periappendiceal abscesses, periappendiceal inflammatory masses, and free air on CT were diagnosed with complicated appendicitis. In addition, intraluminal air, transverse diameter of the appendix, and ascites were identified as important CT findings for predicting complicated appendicitis. C-reactive protein (CRP) level, white blood cell (WBC) count, erythrocyte sedimentation rate (ESR), and body temperature also showed important associations with complicated appendicitis. The AUC, sensitivity, and specificity of the diagnostic algorithm comprising features were 0.91 (95% CI, 0.86–0.95), 91.8% (84.5–96.4), and 90.0% (82.4–95.1) in the development cohort, and 0.7 (0.63–0.84), 85.9% (75.0–93.4), and 58.5% (44.1–71.9) in test cohort, respectively. Conclusion: We propose a diagnostic algorithm based on a decision tree model using CT and clinical findings. This algorithm can be used to differentiate between complicated and noncomplicated appendicitis and to provide an appropriate treatment plan for children with acute appendicitis.

## 1. Introduction

Acute appendicitis is the most common cause of acute abdominal pain. With timely diagnosis, it can be treated with appropriate medical and surgical interventions [1]. However, delayed diagnosis can lead to perforation in 16–39% of adult cases [1]. Perforation and complications are more common in children than in adults. The perforation rate of acute appendicitis was reported to be 80–100% in children younger than three years, while it was approximately 38% in older children [2]. Delayed diagnosis and the presence of complications such as perforation increase the hospitalization period, cost burden, risk of in-hospital infection, morbidity, and mortality [3]. Prompt diagnosis of acute appendicitis is difficult in pediatric patients, and the younger the patient, the more difficult it is to communicate and perform physical examinations cooperatively [4]. Acute appendicitis is misdiagnosed on initial presentation in 28–57% of children younger than 12 years [3]. Even excess CT scan increases the possibility of future malignancy development, so careful handling and minimizing CT scans are more important in children [5]. CT imaging has a high diagnostic accuracy for acute appendicitis [6,7,8,9,10,11]. It is also a good diagnostic imaging modality for identifying the complications of acute appendicitis [1]. Diagnosis of acute perforated appendicitis based on five specific CT findings (abscess, phlegmon, extraluminal air, extraluminal appendicolith, and focal defect in the appendiceal wall) shows overall sensitivity and specificity of 94.9% and 100%, respectively [12]. However, these findings appear in the advanced perforation of the appendix and are not suitable for the detection of early or micro-perforation cases because of reduced clarity [1].

There are several scoring systems to clinically distinguish between pediatric appendicitis patients with simple and perforated appendicitis. In 2008, Andersson et al. developed an appendicitis inflammatory response score composed of clinical signs and symptoms and laboratory tests, including C-reactive protein (CRP) and white blood cell count (WBC) results [13]. In a recent study by Anand et al., hyponatremia due to antidiuretic hormone secretion induced by proinflammatory cytokines such as IL-6 was reported as a predictor of complicated appendicitis [14]. This is useful when imaging techniques such as US or CT are inconclusive or not available [15]. Although there have been studies that predicted complicated and noncomplicated appendicitis based on CT findings in adults [1], to date, no studies have included children. Further, analyzing the clinical elements along with CT images would help improve the diagnostic accuracy in predicting complicated appendicitis. This study aimed to establish a diagnostic algorithm to predict complicated appendicitis in children using CT findings and laboratory and clinical data.

## 2. Theory, Equipment, and Methods

### 2.1. Patient

This single-center, observational, retrospective study was conducted after obtaining approval from the institutional review board. The requirement for informed consent was waived owing to the retrospective nature of the study.

We reviewed the records of 332 consecutive patients aged <18 years who had received a histologic diagnosis of acute appendicitis between January 2014 and December 2018. Among them, 27 were excluded because of the following criteria: unavailable CT images 24 h prior to surgery (*n* = 20), poor quality of CT images for interpretation (*n* = 2), and incomplete medical records (*n* = 5). Among the 315 patients (190 males and 125 females) included in the study, 198 patients who underwent appendectomy between January 2014 and December 2016 were assigned to the development cohort for constructing a diagnostic algorithm and internal validation, and 117 patients who underwent appendectomy between January 2017 and December 2018 were assigned to the test cohort for temporal and external validation.

### 2.2. Laboratory and Clinical Findings

The laboratory and clinical characteristics of the study population were extracted from the laboratory report system and medical records. Inflammatory markers included WBC count, neutrophil count, CRP level, and erythrocyte sedimentation rate (ESR). Clinical findings included duration of symptoms, body temperature, presence of nausea, vomiting, right lower quadrant (RLQ) pain, direct tenderness (DT) of the RLQ, and rebound tenderness (RT) of the RLQ.

The results of the histopathological examination of the surgically resected appendix specimen were collected to categorize acute appendicitis as complicated appendicitis or noncomplicated appendicitis. Complicated appendicitis was defined as gangrenous or perforated appendicitis, and noncomplicated appendicitis was defined as suppurative or inflammatory appendicitis.

### 2.3. CT Acquisition

CT images were acquired using 64- or 128-channel slice multidetector CT scanners (SOMATOM Sensation 64 and SOMATOM Definition Flash CT, Siemens Medical Solutions, Erlangen, Germany). The tube potential (kVp) ranged from 100 to 120 kVp with a CT dose index of 1.44–5.84 mGy, according to the patient’s body weight. An automatic tube current modulation was applied. The other imaging parameters were thickness, 1.5–5 mm; gantry rotation, 0.33 s; pitch, 1.0; and kernel, I30f. All CT images were obtained at the portal venous phase after the administration of an intravenous contrast medium using a clinical weight-based dose (1.5 mL/kg) through a power injector. No oral contrast agent was administered to any patient.

### 2.4. Image Analysis

Axial and coronal reconstruction CT images were retrospectively reviewed on a picture archiving and communication system workstation by two radiologists (HSM and BJ with more than 10 years of experience in the interpretation of pediatric abdominopelvic CT scans) who were blinded to the related clinical information. Any conflicts between the observers’ interpretations were resolved by consensus after the re-evaluation of CT. The following CT findings were evaluated: intraluminal appendiceal air, appendicolith, suspicious wall defects, transverse diameter, periappendiceal fluid, ascites, and periappendiceal infiltration. Specific findings for advanced perforated appendicitis [11], including periappendiceal abscess, periappendiceal inflammatory mass, and free air, were also evaluated. Detailed definitions of each CT finding were as follows—(1) intraluminal appendiceal air: the presence of air within the lumen; (2) intraluminal appendicolith: the presence of well-defined, radiopaque round or oval structures inside the lumen; (3) suspicious wall defect: an interruption in the enhancement of the appendiceal wall (it was judged to be positive only when it was unequivocal and negative when it was difficult to judge); (4) transverse diameter: measurement of maximal short-axis diameter of the inflamed appendix; (5) periappendiceal fluid: poorly defined fluid collection around appendix; (6) ascites: extraluminal fluid attenuation in the abdomen or pelvis with a non-enhancing rim; (7) periappendiceal infiltration: increased attenuation and stranding of the periappendiceal fat; (8) periappendiceal abscess: clearly delineated, discrete fluid collection with rim enhancement; (9) periappendiceal inflammatory mass: diffuse but marked inflammation of the periappendiceal fat; (10) free air: presence of air outside the lumen.

### 2.5. Feature Selection and Constructing Decision Tree Model

A decision tree was used as a machine-learning model to predict complicated appendicitis in children. The advantage of a decision tree is that it is very intuitive and explainable for classification and regression; therefore, it is easy to understand why the results are predicted by the decision tree. In other words, unlike other machine-learning algorithms such as KNN and SVM, Decision Tree has the advantage of using both results and prediction process because of its explainability.

Since periappendiceal abscess, periappendiceal inflammatory mass, and free air were clinically known as direct signs of complicated appendicitis, the decision tree structure that determines complicated appendicitis was constructed at the top of the tree based on the medical doctors’ knowledge. When these three factors were not present, a decision tree for predicting appendicitis was generated using the remaining 18 factors. The static machine-learning toolbox of MATLAB 2022a was used to learn the decision tree, and the grid search method was used to find the optimal hyperparameter of the decision tree. The decision tree in the statistical machine-learning toolbox of MATLAB 2022a provides feature importance for prediction, which computes estimates of feature importance for trees by summing changes in the risk due to splits on every predictor and dividing the sum by the number of branch nodes [16].

### 2.6. Statistical Analysis

Continuous variables are presented as mean ± standard deviation (SD) or as the median and interquartile range (IQR). Categorical variables are expressed as numbers and frequencies. Group comparisons were performed using Student’s t-test or the nonparametric Mann–Whitney U test for continuous variables and the chi-square test or Fisher’s exact test for categorical variables, as appropriate. Interobserver agreement was assessed using intraclass correlation coefficients (ICCs) for continuous data and Cohen’s kappa (κ) for categorical data. An ICC/κ value of ≥0.81 was regarded as excellent agreement, 0.61–0.80 as substantial agreement, 0.41–0.60 as moderate agreement, 0.21–0.40 as fair agreement; and <0.20 as poor agreement.

A decision tree was used to build a diagnostic algorithm to differentiate between noncomplicated and complicated appendicitis.

Sensitivity, specificity, accuracy, and area under the receiver operating characteristic curve (AUC) from the receiver operating characteristic (ROC) curve analysis were calculated to evaluate the diagnostic algorithm for identifying complicated appendicitis. Statistical significance was set at *p* < 0.05. Statistical analyses were performed using SPSS (version 21.0; SPSS, Inc., Chicago, IL, USA). A diagnostic algorithm for identifying complicated appendicitis based on the decision tree algorithm was developed using MATLAB 2022a with Statistical Machine Learning and Deep Learning toolboxes (MathWorks).

## 3. Results

### 3.1. Baseline Character

The study included 315 patients with a mean age of 12.1 ± 3.8 years, and 60.3% (190/315) were male. Among them, 162 showed noncomplicated appendicitis, and 153 showed complicated appendicitis. Table 1 summarizes the age and sex distributions, clinical values on administration, and maximum laboratory results before surgery. The study population was divided into 198 development cohorts and 117 test cohorts according to the date of appendectomy.

### 3.2. Decision Tree Model for Discriminating Complicated Appendicitis

The demographic data, preoperative laboratory results, clinical values, and CT findings between noncomplicated appendicitis and complicated appendicitis in the development cohort are listed in Table 2. All patients with periappendiceal abscesses, periappendiceal inflammatory masses, and free air on CT were diagnosed with complicated appendicitis. All remaining CT findings, including intraluminal appendiceal air, appendicolith, suspicious wall defect, periappendiceal fluid collection, ascites, and periappendiceal infiltration, were significantly more common in complicated appendicitis (*p* < 0.001). The transverse diameter of the appendix was significantly greater in complicated appendicitis. Among the laboratory results, patients with complicated appendicitis had significantly higher WBC and neutrophil counts than those with noncomplicated appendicitis (*p* < 0.001). There were no significant differences in clinical symptoms and signs in terms of RLQ pain, nausea, vomiting, DT/RT in RLQ, symptom duration, and body temperature between noncomplicated appendicitis and complicated appendicitis. The inter-observer agreements of the CT findings are presented in Table A1. We observed substantial to excellent interobserver agreement for the CT findings (Cohen’s κ = 0.73–1, ICC = 0.92). The periappendiceal abscess, periappendiceal inflammatory mass, and free air showed complete agreement between the observers (Cohen’s κ = 1).

**Figure 1:**
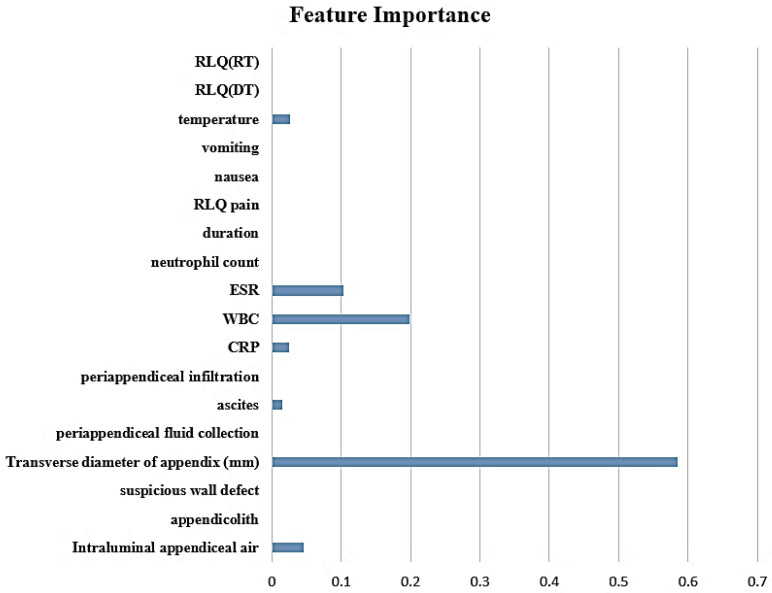
Ranking of feature importance.

**Figure 2:**
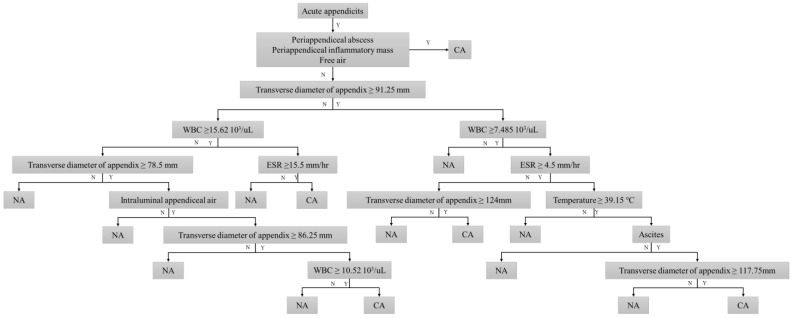
Proposed diagnostic algorithm based on the decision tree to predict complicated appendicitis.

To further identify important features for predicting complicated appendicitis beyond the direct CT findings, we applied the decision tree algorithm. The intraluminal air, transverse diameter of the appendix, ascites, CRP, WBC, ESR, and body temperature were selected as important features for predicting complicated appendicitis (Figure 1). Figure 2 shows the decision tree model for predicting complicated appendicitis.

**Figure 3:**
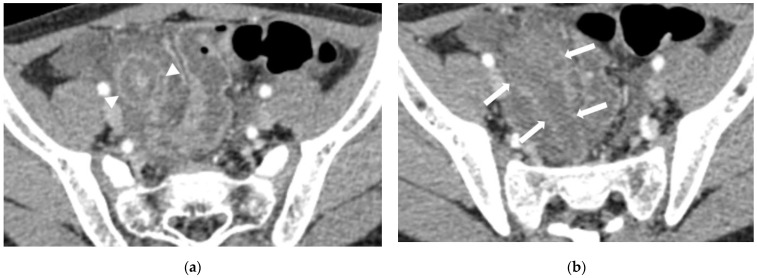
An 8-year-old male with pathologically confirmed complicated appendicitis. (**a**) Distended appendix is seen (arrowheads). (**b**) A 6 cm-sized discrete fluid collection with defined rim enhancement is present adjacent to distended appendix suggesting periappendiceal abscess (arrows).

**Figure 4:**
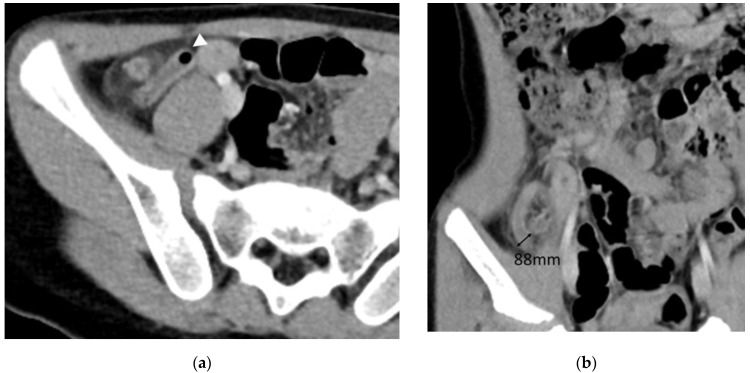
A 6-year-old male with pathologically confirmed complicated appendicitis. (**a**) Axial and (**b**) coronal CT images reveal an 88 mm maximum appendiceal diameter and intraluminal air density (arrowhead). The laboratory test showed increased level of WBC (16.01 × 10^3^/uL).

Patients with a periappendiceal abscess, periappendiceal inflammatory mass, and free air on CT were preferentially predicted to have complicated appendicitis. In addition, a transverse appendix diameter of >91.25 mm was the first partitioning predictor in the decision tree model. Further branching was performed using intraluminal air, ascites, WBC count, ESR, CRP level, and body temperature. These features were used as nodes multiple times with different cutoff values. The decision tree model was able to correctly classify 180 (90.9%) of 198 cases in the development cohort (Table 3). In terms of predicting complicated appendicitis, the sensitivity, and specificity of the decision tree model were 91.8% (95% CI, 0.86–0.95) and 90.0% (82.4–95.1), respectively. The AUC value was 0.91 (0.86–0.95). In the temporal validation cohort, the decision tree model correctly predicted pathological results in 86 of 117 (73.5%) patients, showing moderate discrimination ability with AUC, sensitivity, and specificity of 0.74 (0.63–0.84), 85.9% (75.0–93.4), and 58.5% (44.1–71.9), respectively (Figure 3 and Figure 4).

## 4. Discussion

The current study presents a diagnostic algorithm for diagnosing complicated appendicitis in pediatric patients with high sensitivity and specificity. In children, the diagnosis of complicated appendicitis is more difficult because of indistinctive symptoms and signs compared to those in adults [12]. CT is a well-known accurate imaging modality for the diagnosis of acute appendicitis, with a reported sensitivity of 88–100% and about 95% in adult and pediatric populations, respectively [9,17,18,19,20]. Although US is the first modality of choice for the evaluation of the appendix in pediatric patients, CT is preferred in cases of complicated appendicitis [12]. However, the sensitivity and specificity of CT for diagnosing complicated appendicitis are relatively low, approximately 62% and 81%, respectively [18,21]. The diagnostic algorithm developed in our study showed higher sensitivity and specificity than those previously reported for diagnosing complicated appendicitis.

The proposed diagnostic algorithm includes five CT features to discriminate between complicated and noncomplicated appendicitis. First, complicated appendicitis can be confirmed if the following three findings are present: periappendiceal abscess, periappendiceal inflammatory mass, and free air, which are well-known CT findings of complicated appendicitis in adult patients [12]. Horrow et al. demonstrated that diagnosis of acute perforated appendicitis based on abscess, phlegmon, extraluminal air, extraluminal appendicolith, and focal defect in the appendiceal wall, showed overall sensitivity and specificity of 94.9% and 100%, respectively [12]. Our study evaluated the focal wall defect; however, it did not appear to be a significant factor in the diagnostic algorithm. A focal wall defect is a CT feature that can be evaluated only when the contrast-enhanced CT scan is performed well enough to clearly identify the walls of the appendix [12]. In pediatric patients, the use of contrast medium is less favorable than in adults, and it is difficult to achieve a successful examination with an appropriate enhancement time. Therefore, focal wall defects may not be valuable in the algorithm for pediatric patients. Similarly, the evaluation of phlegmon, which represents a periappendiceal inflammatory mass in our study, can greatly contribute to the diagnosis of complicated appendicitis. If contrast enhancement is not appropriate, it may be difficult to identify an abscess with an enhanced wall; however, inflammation around the appendix can still be identified [12].

In our study, a transverse appendix diameter of >91.25 mm was the first partitioning predictor with the greatest feature importance in the decision tree model. The normal appendix diameter is ≤6 mm [22,23], and >6 mm suggests acute appendicitis with approximately 93% sensitivity [24] and 92% specificity [24]. According to a previous study [25], the cutoff value for appendix diameter in acute appendicitis and its association with perforation was approximately 9.25 mm in adult patients. In younger children, the diameter of the appendix may vary depending on age; however, the diameter remains relativity constant beyond the age of 6–7 years [26,27,28]. The average age of the patients in our study was approximately 12 years; therefore, the standard for the appendix diameter could be substituted with the same value as for adults. Considering this, the numerical value of the diameter of the appendix divided by complicated appendicitis in our research algorithm corresponds to the results of previous studies [25].

Intraluminal air was the second most important CT feature in the decision tree model. Intraluminal air in the appendix has been controversial in several previous studies [29,30]. The interpretation of intraluminal air depends on whether there is inflammation in the appendix. In a normal appendix, intraluminal air is related to communication with the cecal lumen. However, in an inflamed appendix, intraluminal air is associated with infection [29]. Intraluminal air within an obstructed appendix is a significant predictive risk factor for necrosis or perforation of the appendix [29]. Since our study included patients with appendicitis, the results indicate a frequent association of intraluminal air with complicated appendicitis.

Our study demonstrated that WBC count, CRP level, ESR, temperature, and ascites were clinical factors that could differentiate complicated appendicitis in the decision tree model. In the lower branch of the decision tree model, these clinical factors can be used to diagnose complicated appendicitis in several steps. Elevated CRP and WBC levels indicate advanced appendicitis in children, with optimal cutoff values of 14,000/mm^3^ and 3.9 mg/dL, respectively [31]. In our algorithm, the cutoff value of WBC was 15,620/mm^3^, which is slightly higher than the previous result as the third partitioning step. Increased CRP is a good indicator of appendiceal perforation or abscess formation in adult patients [32]. CRP levels increase in such cases of prolonged inflammation, even when the WBC count does not increase [33]. Further, Chung et al. reported that CRP level was a good indicator of appendiceal perforation or abscess formation in children and suggested a cutoff serum CRP level of 5 mg/dL for the diagnosis of perforated appendicitis [34]. In our algorithm, the cutoff value of CRP was 2.05 mg/L, which is lower than the previous result. A histological evaluation of 264 perforated appendicitis cases in children identified a high WBC count (>13.5 × 10^9^/L) and ESR (>15.0 mm/hr) as independent predictors [35]. Our algorithm also demonstrated elevated ESR as a discriminating feature with a cutoff of 4.5 mm/hr, which is lower than that reported previously. We presume that the reason for the low cutoff values of CRP and ESR in our algorithm is that other factors with higher importance were considered in advance in the algorithm tree.

Recent studies have demonstrated hyponatremia could be a novel predictor for complicated appendicitis [14,36]. However, we found there is no significant difference in sodium levels between complicated and noncomplicated appendicitis in our development cohorts (complicated appendicitis, mean level, 138.0 ± 2.3 mEq/L, range 133–144 mEq/L vs. noncomplicated appendicitis, 138.1 ± 2.0, range 134–142 mEq/L; *p*-value = 0.68). Although antidiuretic hormone released by proinflammatory cytokines such as IL-6 induces hyponatremia, serum sodium levels could rise due to many variable factors, such as dehydration, vomiting, extreme diarrhea, or fever. We believe that other confounding factors that occurred in the cohort affected serum sodium.

The exclusion of complicated appendicitis can affect the treatment choice. Nonsurgical treatment of noncomplicated acute appendicitis in children is an alternative to appendectomy. A previous systematic review and meta-analysis showed that complications and length of hospital stay were similar between patients treated with antibiotics and those who underwent appendectomy. Nonsurgical treatment of noncomplicated acute appendicitis is safe and efficient in pediatric patients [37]. On the other side, complicated appendicitis with abscess or phlegmon treated by a delayed appendectomy followed by drainage should be considered first [38].

## 5. Conclusions

In this paper, we proposed a diagnostic algorithm based on a decision tree model using CT findings and clinical features, which yielded high diagnostic performance in both the development and test cohorts. This algorithm could be used to differentiate between complicated and noncomplicated appendicitis and provide an appropriate treatment plan for children with acute appendicitis.

In spite of our contributions, our study had several limitations. First, there were certain limitations due to its retrospective design. The electronic medical records for clinical symptoms and signs were not standardized, and the data for some variables were unclear. This may have resulted in clinical symptoms and signs not being selected as important predictive features of complicated appendicitis in this study. In addition, we included patients who had undergone appendectomy and pathologically proven acute appendicitis instead of consecutive patients who were clinically suspected of having acute appendicitis. Furthermore, it may be ideal to analyze not only CT but also USG. However, because there was some limitation in the reliable reinterpretation of already captured USG images in the retrospective design, we chose only an analysis of CT imaging. Thus, a prospective design with a clinical diagnosis of acute appendicitis patients using various diagnostic modalities, including CT or USG, with thorough history taking may have yielded more conclusive results. Second, we adopted the surgical pathologic results as the final diagnosis of the type of appendicitis. In some cases, the pathology specimen may not have included a perforated site, therefore leading to a false-negative diagnosis of complicated appendicitis [1]. To avoid misdiagnosis, surgical findings should be considered. However, it was not possible to collect surgical findings from this retrospective study. Third, although we validated the diagnostic performance of the algorithm in a temporally independent test cohort, the development and test cohorts were obtained from the same institution. Thus, future studies that perform external validation in a multicenter setting with different populations are needed. Fourth, limited laboratory data were evaluated. A recent study has investigated hyponatremia in patients with complicated appendicitis [14]. They reported that hyponatremia played a role in predicting complicated appendicitis. This may be explained by the role of proinflammatory cytokines such as IL-6 in the non-osmotic release of antidiuretic hormone [14,36]. However, we focused on the criteria of “appendicitis inflammatory response (AIR) score” and included traditional inflammatory laboratory markers. So future studies including various laboratory results should be performed. Finally, we did not evaluate outcome results regarding the disease course between complicated appendicitis and noncomplicated appendicitis.

## Figures and Tables

**Table 1:** Baseline characteristics of the study population.

Characteristic	All (*n* = 315)	Development Cohort (*n* = 198)	Test Cohort (*n* = 117)	*p*-Value
Age(years) †	12.1 ± 3.8	12.1 ± 3.8	12.1 ± 4.0	0.665
Sex				
Male	190 (60.3%)	124 (62.6%)	66 (56.4%)	0.3
Female	125 (39.7%)	74 (37.4%)	51 (43.6%)	
CT findings				
Periappendiceal abscess	6 (1.9%)	3 (1.5%)	3 (2.6%)	0.67
Periappendiceal inflammatory mass	21 (6.7%)	14 (7.1%)	7 (6.0%)	0.71
Free air	7 (2.2%)	5 (2.5%)	2 (1.7%)	1.00
Intraluminal appendiceal air	82 (26.0%)	55 (27.8%)	27 (23.1%)	0.36
Appendicolith	135 (42.9%)	85 (42.9%)	50 (42.7%)	1.00
Suspicious wall defect	63 (20.0%)	48 (24.2%)	15 (12.8%)	0.02
Transverse diameter of appendix (mm) †	100.6 ± 31.1	98.3 ± 30.4	102.5 ± 31.9	0.89
Periappendiceal fluid collection	71 (22.5%)	49 (24.7%)	22 (18.8%)	0.22
ascites	185 (58.7%)	128 (64.6%)	57 (48.7%)	0.01
Periappendiceal infiltration	240 (76.2%)	158 (79.8%)	82 (70.1%)	0.05
Laboratory results				
CRP (mg/L) †	19.5 ± 35.8	20.7 ± 38.9	18.4 ± 32.2	0.07
WBC (10^3^/uL) †	3.2 ± 4.8	13.3 ± 4.6	13.2 ± 4.7	0.77
Neutrophil count (/uL) †	10,591.7 ± 4682.2	10,612.0 ± 4543.8	10,804.5 ± 4412.9	<0.01
ESR (mm/hr) †	15.10 ± 14.400	14.3 ± 14.6	16.0 ± 13.3	0.27
Clinical Symptom and sign				
RLQ pain	142 (45.1%)	81 (40.9%)	61 (52.1%)	0.05
Nausea	130 (41.3%)	88 (44.4%)	42 (35.9%)	0.14
Vomiting	146 (46.3%)	94 (47.5%)	52 (44.4%)	0.60
RLQ(DT)	265 (84.1%)	166 (83.8%)	99 (84.6%)	0.86
RLQ(RT)	107 (34.0%)	73 (36.9%)	34 (29.1%)	0.16
Duration (hr) †	21.5 ± 23.2	21.9 ± 24.2	17.9 ± 17.9	0.759
Temperature(°C) †	37.1 ± 0.7	37.0 ± 0.7	37.2 ± 0.8	0.955
Pathologic results				
Noncomplicated appendicitis	138 (43.8%)	98 (49.5%)	40 (34.2%)	0.01
Complicated appendicitis	177 (56.2%)	100 (50.5%)	77 (65.8%)	

Note—data in parentheses are percentages. † Data values are presented as a mean ± standard deviation.

**Table 2:** Comparison between Noncomplicated and complicated appendicitis in development cohort.

	Noncomplicated Appendicitis (*n* = 98)	Complicated Appendicitis (*n* = 100)	*p*-Value
Age (years)	12.1 ± 3.6	12.1 ± 3.9	0.94
Sex			
Male	68 (69.4%)	56 (56.0%)	0.05
Female	30 (30.6%)	44 (44.0%)	
CT findings			
Periappendiceal abscess	0 (0.0%)	3 (3.0%)	0.25
Periappendiceal inflammatory mass	0 (0.0%)	14 (14.0%)	<0.001
Free air	0 (0.0%)	5 (5.0%)	0.06
Intraluminal appendiceal air	38 (38.8%)	17 (17.0%)	<0.001
Appendicolith	24 (24.5%)	61 (61.0%)	<0.001
Suspicious wall defect	5 (5.1%)	43 (43.0%)	<0.001
Transverse diameter of appendix (mm)	83.0 ± 22.8	117.1 ± 28.5	<0.001
Periappendiceal fluid collection	8 (8.2%)	41(41.0%)	<0.001
Ascites	46 (46.9%)	82 (82.0%)	<0.001
Periappendiceal infiltration	62 (63.3%)	96 (96.0%)	<0.001
Laboratory results			
CRP (mg/L) †	11.7 ± 15.0	29.7 ± 51.3	0.12
WBC (10^3^/uL) †	11.2 ± 4.4	15.4 ± 4.4	<0.001
Neutrophil count † (/uL)	8516.7 ± 4223.4	12,757.4 ± 4397.9	<0.001
ESR (mm/hr) †	12.51 ± 13.799	16.74 ± 15.952	0.06
Clinical Symptom and sign			
RLQ pain	40 (40.8%)	41 (41.0%)	0.98
Nausea	47 (48.0%)	41 (41.0%)	0.32
Vomiting	48 (49.0%)	46 (46.0%)	0.67
RLQ(DT)	84 (85.7%)	82 (82.0%)	0.48
RLQ(RT)	34 (34.7%)	39 (39.0%)	0.53
Duration (hr) †	23.7 ± 26.7	22.8 ± 24.8	0.79
Temperature (°C) †	37.0 ± 0.7	37.0 ± 0.7	0.97

Note—data in parentheses are percentages. † Data values are presented as a mean ± standard deviation.

**Table 3:** Diagnostic performance of decision tree algorithm for diagnosing complicated acute appendicitis.

			Development Group		Test Group	
		Pathology Results	Complicated Appendicitis	Noncomplicated Appendicitis	Complicated Appendicitis	Noncomplicated Appendicitis
**Model** **prediction**	Complicated appendicitis		90	10	55	22
	Noncomplicated appendicitis		8	90	9	31
**AUC**			0.91 (0.86, 0.95)		0.74 (0.63, 0.84)	
**Sensitivity (%)**			91.8 (84.5, 96.4)		85.9 (75.0, 93.4)	
**Specificity (%)**			90.0 (82.4, 95.1)		58.5 (44.1, 71.9)	
**Accuracy (%)**			90.9 (86.0, 94.5)		73.5 (64.2, 81.2)	
**PPV (%)**			90 (83.3, 94.2)		71.4 (64.2, 77.8)	
**NPV (%)**			91.8 (85.2, 95.6)		77.5 (64.3, 86.8)	
**PLR**			9.2 (5.1, 16.6)		2.1 (1.5, 2.9)	
**NLR**			0.1 (0.1, 0.2)		0.2 (0.1, 0.5)	

Note—data in parentheses are 95% confidence intervals. PPV, Positive predictive value; NPV, Negative predictive value; PLR, positive likelihood ratio; NLR. Negative likelihood ratio.

## Data Availability

Not applicable.

## Appendix A

**Table A1:** Inter-observer agreement for CT findings.

	Periappendiceal Abscess	Periappendiceal Inflammatory Mass	Free Air	Intraluminal Appendiceal Air	Appendicolith	Suspicious Wall Defect	Transverse Diameter of Appendix (mm) †	Periappendiceal Fluid Collection	Ascites	Periappendiceal Infiltration
Kappa	1	1	1	0.98	0.97	0.88	0.92	0.87	0.94	0.87
95% CI	1.00, 1.00	1.00, 1.00	1.00, 1.00	0.95, 1.00	0.95, 1.00	0.80, 0.94	0.90, 0.93	0.80, 0.94	0.90, 0.98	0.80, 0.93

Note—Unless otherwise specified, a Cohen’s κ was used for evaluating inter-reader agreement. † Intraclass correlation coefficient was used for evaluating inter-reader agreement.
